# Treatment of Calcified Peripheral Artery Disease With the Shockwave Forward Peripheral Intravascular Lithotripsy System: One-Year Outcomes From the FORWARD PAD IDE and Mini S Feasibility Studies

**DOI:** 10.1016/j.jscai.2026.105579

**Published:** 2026-08-27

**Authors:** Andrew Holden, John D. Corl, Sameh Sayfo, Bibombe P. Mwipatayi, Manar Khashram, Nicolas W. Shammas, Anderson Mehrle, Anvar Babaev, Peter A. Soukas, Richard Pin, Padma Seethamraju, Nick E.J. West, Daniel Clair

**Affiliations:** a Department of Interventional Radiology, Auckland City Hospital, Auckland, New Zealand; b Department of Interventional Cardiology, The Lindner Center for Research and Education at The Christ Hospital, Cincinnati, Ohio; c Department of Interventional Cardiology, Baylor Scott & White The Heart Hospital – Plano, Plano, Texas; d Department of Vascular Surgery, Royal Perth Hospital, Perth, Australia; e Department of Vascular Surgery, Waikato District Hospital, Hamilton, New Zealand; f Midwest Cardiovascular Research Foundation, Davenport, Iowa; g Department of Cardiology, Ascension St. John Jane Phillips Medical Center, Bartlesville, Oklahoma; h Department of Medicine, NYU Langone Medical Center, New York, New York; i Department of Medicine, The Miriam Hospital – Alpert Brown Medical School, Providence, Rhode Island; j Department of Vascular Surgery, Southcoast Health Heart & Vascular, Dartmouth, Massachusetts; k Shockwave Medical Inc, Santa Clara, California; l Department of Vascular Surgery, Vanderbilt University Medical Center, Nashville, Tennessee

**Keywords:** calcified lesions, forward intravascular lithotripsy, peripheral artery disease

Severe calcification, chronic total occlusions (CTOs), and medial calcification are obstacles for endovascular treatment of peripheral artery disease and contribute to high mortality and amputation rates. In complex patients, current calcium modification technologies can be limited by an inability to deliver across severely stenosed lesions and associated risks of vessel damage and distal embolization. The novel Javelin intravascular lithotripsy (IVL) catheter (Shockwave Medical)^1^ was designed to safely modify calcification in extremely narrow crossing channels where a guide wire can be passed (Figure 1). The 1-year outcomes of the Forward-Shifted Intravascular Lithotripsy (IVL) Technology in a Prospective, Multi-center, Single-arm Investigational Device Exemption (FORWARD PAD IDE) Study and Feasibility studies are reported here.

**Figure 1:**
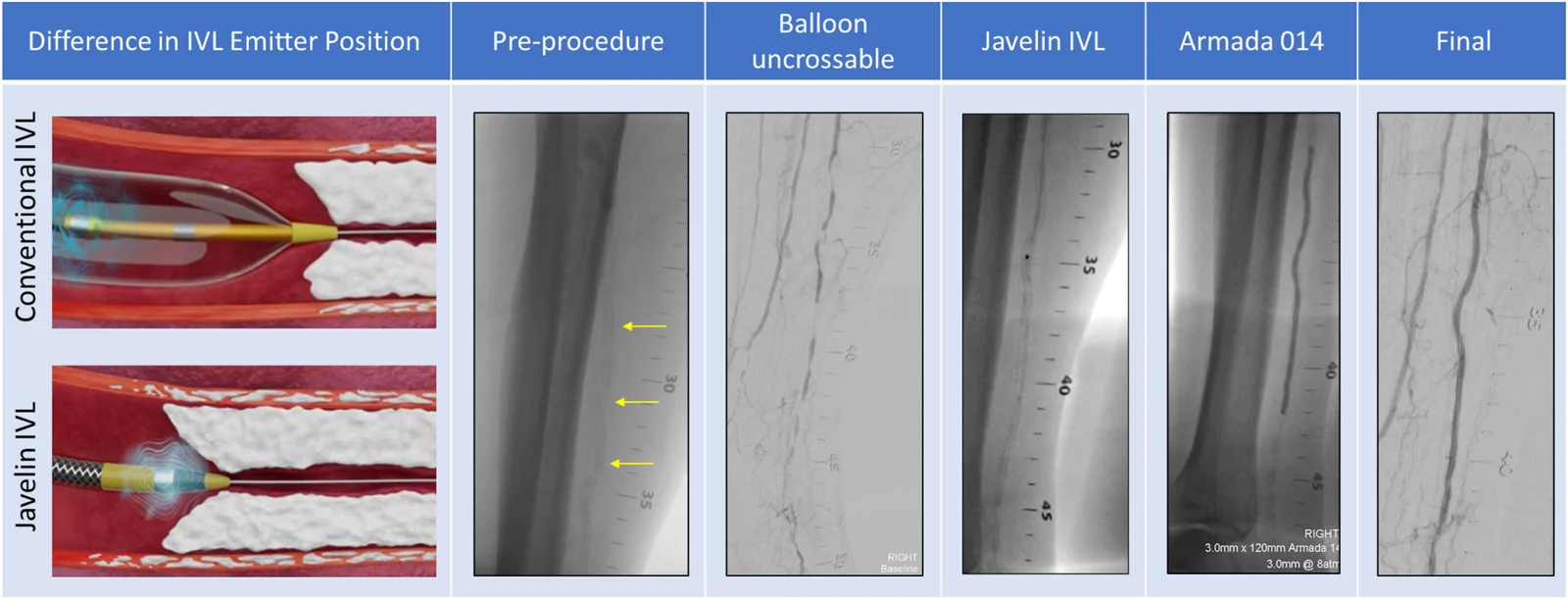
**The Javelin intravascular lithotripsy (IVL) catheter has a single distally positioned IVL emitter within a small chamber close to the tip, which replaces the balloon in conventional IVL devices.** Therefore, acoustic energy can be delivered at the tip of the catheter, approximated to balloon-uncrossable or difficult-to-cross calcified lesions. In the example case, an 85-year-old man presented with right foot pain and tissue loss over the metatarsals. Angiography revealed severe intimal and medial calcification in the posterior tibial artery. After crossing the lesion with a 0.014-inch guide wire, other devices were unable to be passed through the lesion. Javelin IVL was used to modify and cross the length of the lesion, which was subsequently optimized with balloon-after postdilation. The patient subsequently had resolution of rest pain, tissue healing, and sustained patency through 12 months. Case courtesy of Andrew Holden, Auckland City Hospital, New Zealand.

In the prospective, single-arm Mini S Feasibility (NCT05058456) and FORWARD PAD IDE (NCT05858905) studies, 110 patients with 124 core laboratory-assessed lesions (128 site reported) were enrolled. The study had an independent core laboratory and clinical events committee (Yale Cardiovascular Research Group). Written informed consent was obtained prior to study-specific requirements, and all sites were required to follow local legal and regulatory requirements for ethics committee and institutional review board approvals. Major adverse events, cardiovascular-related death, clinically driven target lesion revascularization (CD-TLR), and unplanned target limb major amputation were assessed at 12 months. For above-the-knee (ATK) lesions, primary patency was freedom from ≥50% diameter stenosis determined by duplex ultrasound (DUS) and freedom from any CD-TLR. For below-the-knee (BTK) lesions, primary patency was freedom from total occlusion by DUS and freedom from any CD-TLR. Primary-assisted patency was defined as achieving the diameter stenosis (ATK: ≥50%; BTK: 100%, by DUS), including the use of additional or secondary procedures as long as the target lesion was not completely occluded.^2^ Secondary patency was defined as achieving a diameter stenosis (ATK: ≥50%; BTK: 100%, by DUS), including any secondary procedures inclusive of those for total occlusions.^2^

There were 10 deaths, 4 withdrawals, and 3 patients were lost to follow-up after follow-up visits were completed. Of the 98 eligible patients for their 12-month visit, follow-up compliance was 94.9% (93/98). Patient characteristics have been previously published^1^ and are in the Supplemental Material; core laboratory-assessed lesion characteristics included 83.9% (104/124) with severe calcification following Peripheral Academic Research Consortium definitions, diameter stenosis of 83.0% ± 17.4% (n = 120), and CTOs of 41.7% (50/120). Site-reported procedural measures included 4.7% (6/128) with predilation, 8.6% (11/128) with >1 Javelin catheter used, 29.7% (38/128) with commercial balloon-based IVL use after Javelin, 42.2% (54/128) with drug-coated balloon use, and 21.1% (27/128) with a stent implanted. At final angiography, patients had an acute gain of 2.8 ± 1.2 mm (n = 117), residual stenosis of 23.1% ± 8.6% (n = 119), and only 1 type D dissection. The 12-month overall rate of major adverse events was 18.6% (19/102), with 3.9% (4/102) of subjects experiencing cardiovascular-related death, 1.0% (1/102) with major amputation, and 14.7% (15/102) having CD-TLR. The 4 cardiovascular-related deaths were adjudicated as unrelated to the procedure or the study device by the clinical events committee. Primary, primary-assisted, and secondary patency in ATK lesions were 72.7% (40/55), 74.5% (41/55), and 76.4% (42/55) and in BTK lesions were 61.5% (24/39), 78.4% (29/37), and 88.6% (31/35), respectively.

The combination of calcification severity, degree of stenosis, and presence of CTOs in the FORWARD PAD IDE/Feasibility cohort were more complex than those included in prior clinical trials.^3^^,^^4^ Despite the challenging patient population, stenosis reduction, and acute gains were consistent with the prior balloon-based IVL experience.^3^ The calcium modification was also achieved without perforations or embolization^1^ that have been reported with atherectomy.^4^ Crosstrial comparisons are limited given differences in the study design, lesion complexity, the degree of calcification, and patient populations. However, given the lack of a control arm in the FORWARD PAD IDE and Mini S Feasibility studies, in context with other trials,^4^^,^^5^ the 1-year patency, amputation, and revascularization rates appear encouraging.

Initial experience with Javelin IVL suggests a primary role for the technology in the modification of vascular calcification in difficult-to-cross or balloon-uncrossable lesions. This enables crossing or facilitates subsequent crossing of other devices, including postdilation balloons, to optimize treatment results. Ongoing clinical use of Javelin IVL will determine how this novel IVL platform will influence current calcium modification algorithms for both ATK and BTK lesions.

## CRediT authorship contribution statement

**Andrew Holden:** Writing – review & editing, Writing – original draft, Supervision, Investigation, Formal analysis, Data curation. **John D. Corl:** Writing – review & editing, Writing – original draft, Supervision, Formal analysis, Data curation. **Sameh Sayfo:** Writing – review & editing, Data curation. **Bibombe P. Mwipatayi:** Writing – review & editing, Data curation. **Manar Khashram:** Writing – review & editing, Data curation. **Nicolas W. Shammas:** Writing – review & editing, Data curation. **Anderson Mehrle:** Writing – review & editing, Data curation. **Anvar Babaev:** Writing – review & editing, Data curation. **Peter A. Soukas:** Writing – review & editing, Data curation. **Richard Pin:** Writing – review & editing, Data curation. **Padma Seethamraju:** Writing – review & editing, Validation, Methodology, Formal analysis, Conceptualization. **Nick E.J. West:** Writing – review & editing, Writing – original draft, Supervision, Methodology, Investigation, Formal analysis. **Daniel Clair:** Writing – review & editing, Writing – original draft, Supervision, Data curation.

## Declaration of competing interest

Andrew Holden is on the speaker’s bureau and advisory board for Shockwave Medical Inc. John D. Corl is on the advisory board for Shockwave Medical Inc. Nicolas Shammas receives research and educational grants from Bard, Abbott, Philips, AngioDynamics, and Boston Scientific. Peter Soukas is a consultant and advisory board member for Shockwave Medical Inc. Padma Seetharaju and Nick E.J. West are employees of Shockwave Medical Inc. Daniel Clair is on the advisory board for Shockwave Medical Inc. All other authors declared no potential conflicts of interest with respect to the research, authorship, and/or publication of this article.

## Footnotes

To access the supplemental material accompanying this article, visit the online version of the *Journal of the Society for Cardiovascular Angiography & Interventions* at 10.1016/j.jscai.2026.105579.
