# Supplementary material for: Treatment of Calcified Peripheral Artery Disease With the Shockwave Forward Peripheral Intravascular Lithotripsy System: One-Year Outcomes From the FORWARD PAD IDE and Mini S Feasibility Studies
Source: J Soc Cardiovasc Angiogr Interv. 2026 Aug 27;5(10):105579. doi: 10.1016/j.jscai.2026.105579 (PMC13700031; doi:10.1016/j.jscai.2026.105579)
Supplement: Supplementary file 1 [file mmc1.docx]

Supplemental Material: Baseline patient and lesion characteristics from the FORWARD PAD IDE and Feasibility Studies

| **Baseline Medical History** | **FORWARD PAD** |
| --- | --- |
| Hypertension | 93.6% (103/110) |
| Hyperlipidemia | 84.5% (93/110) |
| Smoking | 67.3% (74/110) |
| Diabetes mellitus | 58.2% (64/110) |
| Myocardial Infarction | 26.4% (29/110) |
| Coronary Artery Disease | 53.6% (59/110) |
| Coronary Stenting or Bypass Procedure | 44.5% (49/110) |
| Renal Insufficiency | 18.2% (20/110) |
| CVA or TIA | 10.9% (12/110) |
| Atrial Fibrillation | 22.7% (25/110) |
| Respiratory/Pulmonary Disease | 33.6% (37/110) |
| Cancer (Malignant or Non-malignant) | 26.4% (29/110) |
| Hospitalized within the Last 60 Days | 13.6% (15/110) |
| Prior Peripheral Procedures | 46.4% (51/110) |

| **Baseline Lesion Angiographic Measurements** | **FORWARD PAD** |
| --- | --- |
| Lesion Location |  |
| Common Femoral Artery | 1.6% (2/124) |
| Sup. Femoral Artery | 40.3% (50/124) |
| Popliteal Artery (Above the Knee) | 14.5% (18/124) |
| Popliteal Artery (Below the Knee) | 8.1% (10/124) |
| Anterior Tibial Artery | 13.7% (17/124) |
| Tibio-Peroneal Trunk | 8.1% (10/124) |
| Peroneal Artery | 5.6% (7/124) |
| Posterior Tibial Artery | 8.1% (10/124) |
| Number of Target Lesions |  |
| 1 | 88.7% (110/124) |
| 2 | 11.3% (14/124) |
| BTK Lesion | 43.5% (54/124) |
| Calcification by PARC |  |
| None/Mild | 4.8% (6/124) |
| Moderate | 11.3% (14/124) |
| Severe | 83.9% (104/124) |
| Total Length of Ca (mm) | 130.1 ± 90.3 (111) |
| Lesion Length (mm) | 78.4 ± 62.2 (123) |
| Long Lesion (≥150 mm) | 13.0% (16/123) |
| MLD | 0.7 ± 0.7 (120) |
| RVD | 4.2 ± 1.4 (120) |
| Diameter Stenosis | 83.0 ± 17.4 (120) |
| Chronic Total Occlusion | 41.7% (50/120) |
